# Supplementary material for: Genetic architecture of routinely acquired blood tests in a British South Asian cohort
Source: Nat Commun. 2024 Oct 16;15:8929. doi: 10.1038/s41467-024-53091-x (PMC11484750; doi:10.1038/s41467-024-53091-x)
Supplement: Supplementary file 1 — Supplementary Information [file 41467_2024_53091_MOESM1_ESM.pdf]

Supplementary materials for "Genetic architecture of routinely acquired blood tests in a British South Asian cohort"

Overview of phenotype quality control

An overview of the phenotype quality control pipeline is shown in the figure below.

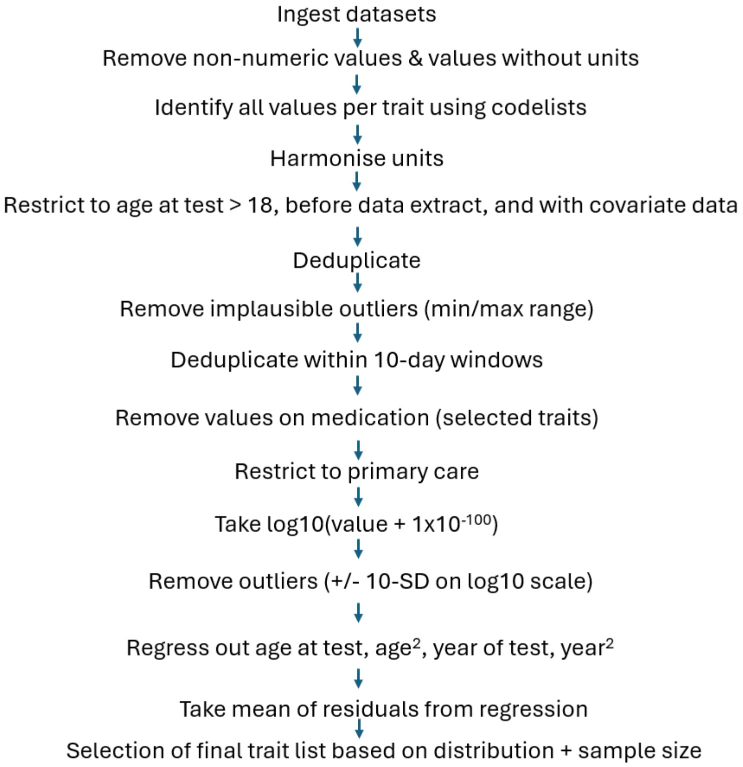

Supplementary figure 1: overview of phenotype quality control.

Primary vs secondary care data

Overall there was a strong correlation between trait values obtained in primary and secondary care (supplementary figure 2), however primary care traits tended to have a lower variance than in secondary care, presumably a reflection of fluctuations in blood test parameters due to acute illness (supplementary figure 3). In general the agreement between primary care and secondary care values was excellent. The clearest discrepancies were for acute-phase reactants such as CRP, ferritin, and white blood cell count, which tended to be higher in the secondary care dataset, as expected given that this dataset contains data from acutely unwell inpatients.

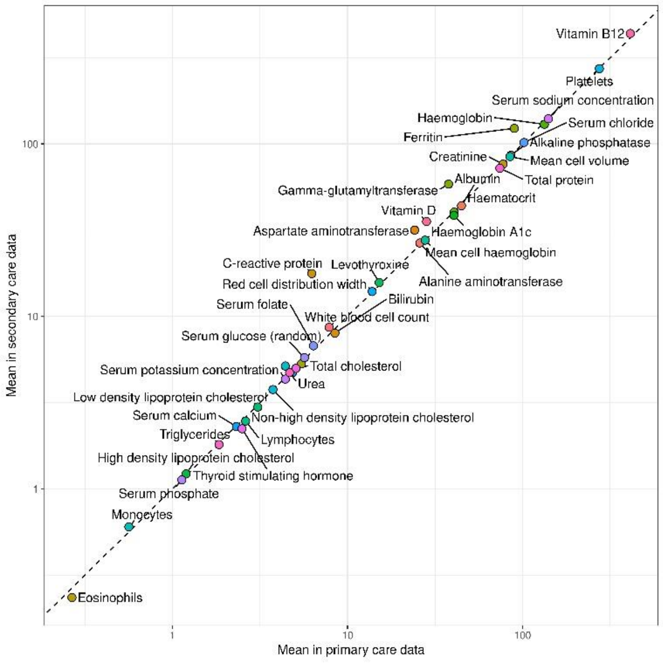

Supplementary figure 2: Scatter plot showing the relationship between primary care and secondary care data per-individual means. Note the log10-axes.

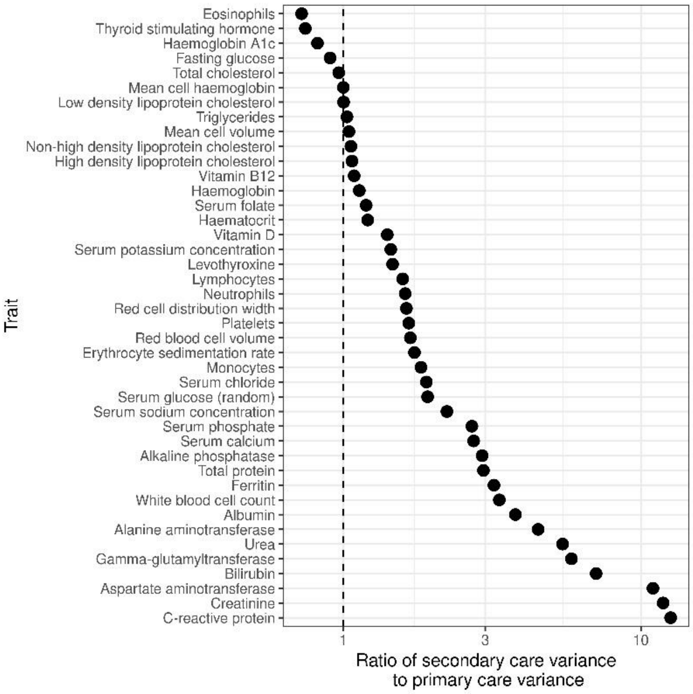

Supplementary figure 3: Forest plot showing the ratio of secondary care variance to primary care variance for each trait. For most traits, there was greater variation in the secondary care dataset, likely reflecting fluctuations in blood test markers of acute illness.

Observed trait distributions

Following quality control the observed trait distributions largely followed a normal distribution on the log-10 scale with the exception of eosinophils, which were right-skewed, and the acute phase reactants ESR and CRP, which were bimodal (supplementary figure 4).

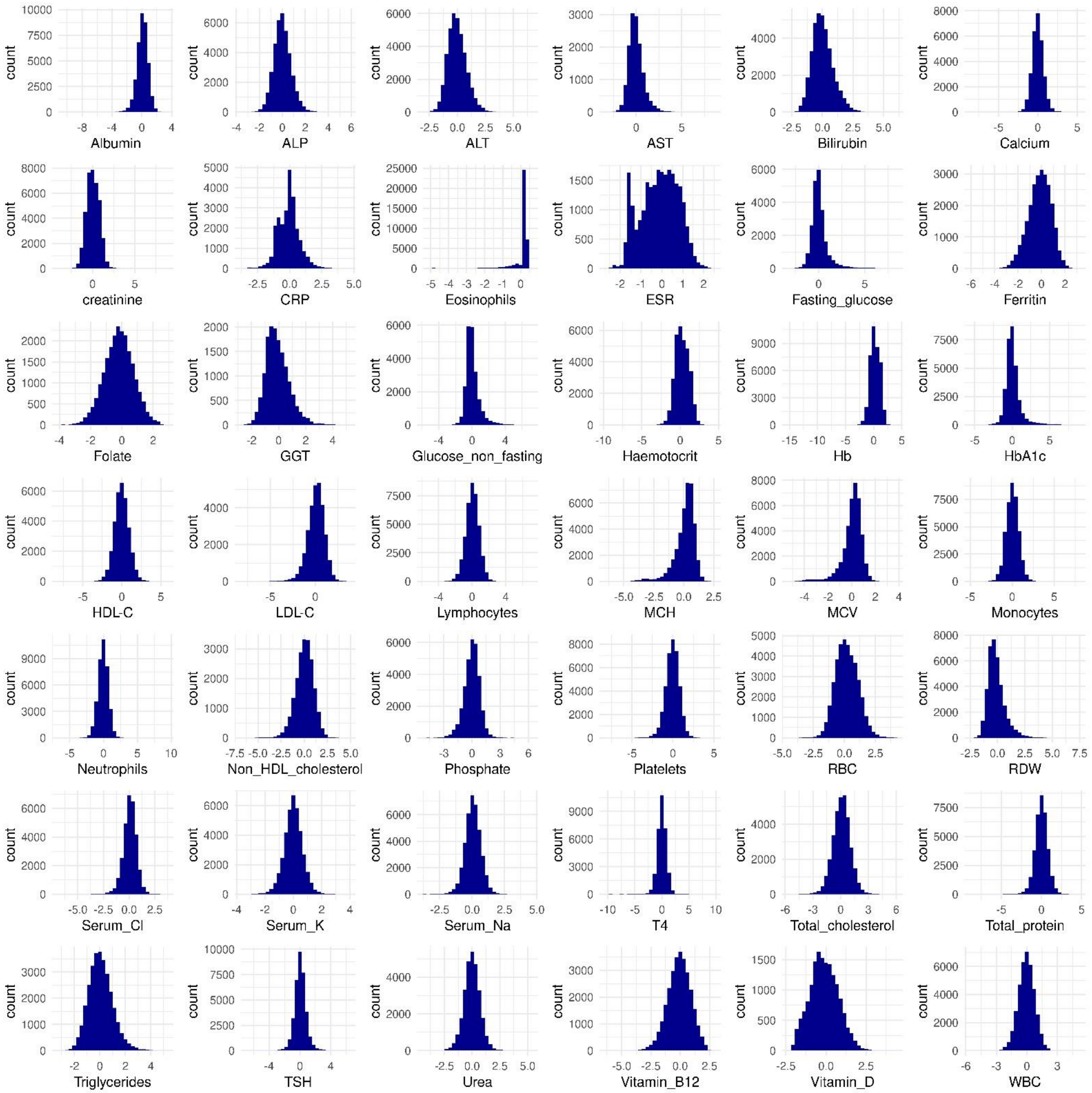

Supplementary figure 4: Histograms of quantitative traits on the log-10 scale following quality control.

Intra-individual variation

To explore the extent of intra-individual variation, we considered the proportion of individuals whose intra-individual variance exceeded that of the population variance for each trait. In general this proportion was low, with >80% of participants having lower intra-individual variation than the population-level variation.

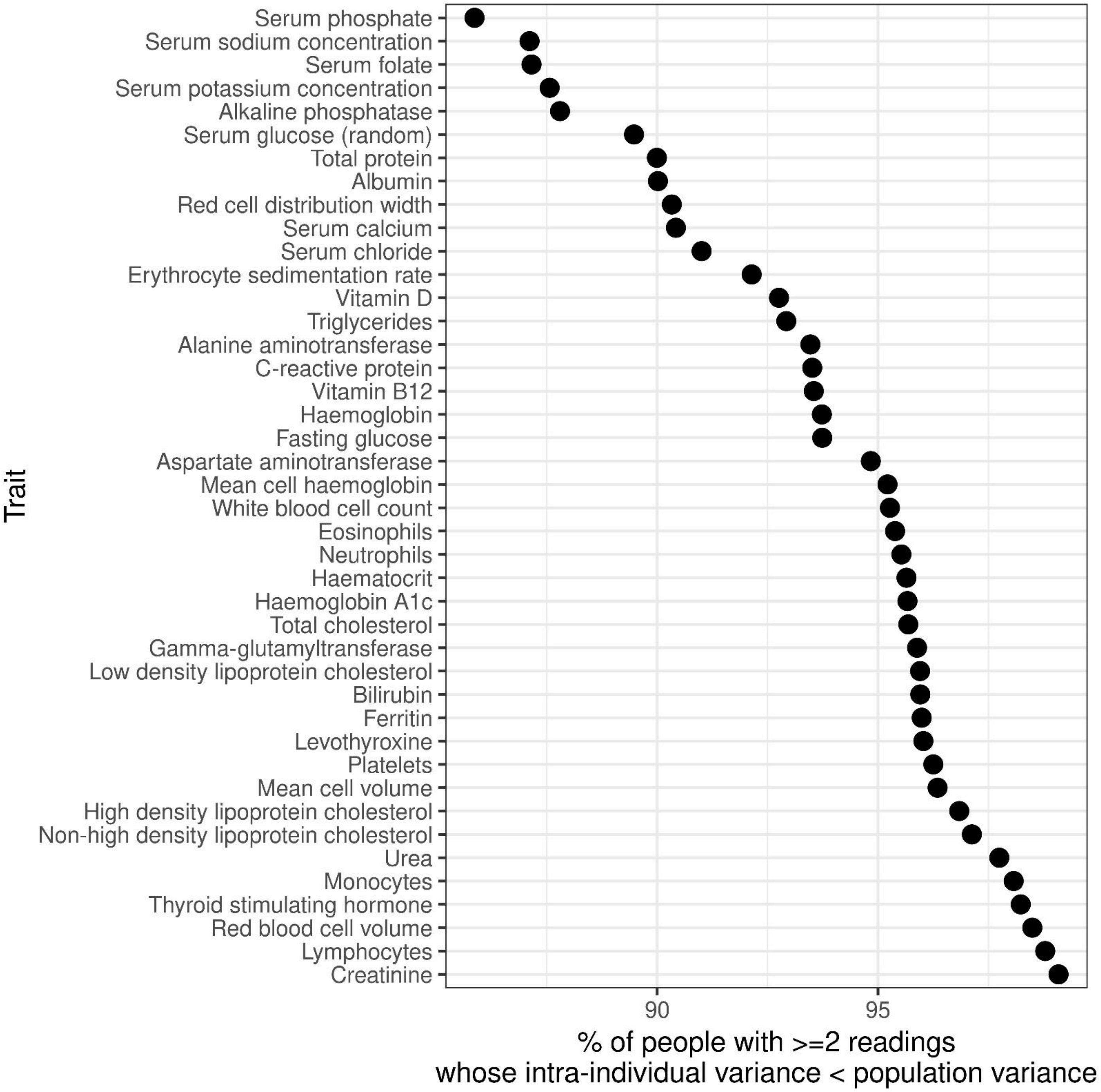

Supplementary figure 5: Forest plot showing the % of people with lower intra-individual variance than population variance for each trait.

Influence of confounders

We explored the variance explained in each trait by several putative confounders using linear models. As expected, data source, year of acquisition, age, and gender all had an impact on the traits examined. Gender had the strongest impact for known sex-dimorphic traits such as creatinine and red cell volume.

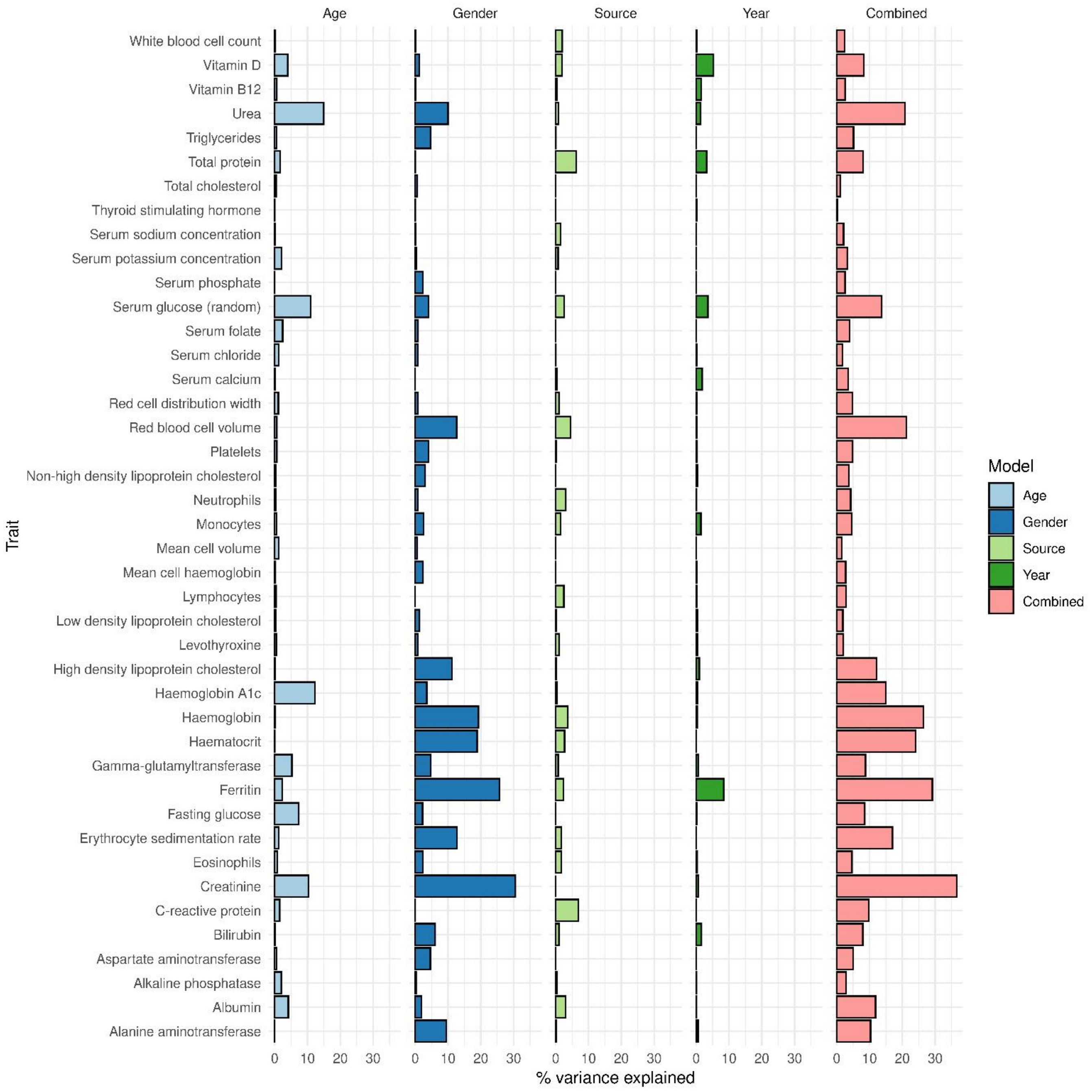

Supplementary figure 6: Barplots showing the variance explained (%) by selected confounding variables using raw unique timepoint data (i.e. including multiple readings per person). The combined model was of the form trait ~ age + gender + source + year. Traits were rank-inverse-normalised prior to model fit.
