# Supplementary material for: Genetic architecture of routinely acquired blood tests in a British South Asian cohort
Source: Nat Commun. 2024 Oct 16;15:8929. doi: 10.1038/s41467-024-53091-x (PMC11484750; doi:10.1038/s41467-024-53091-x)
Supplement: Supplementary file 3 — Description of Additional Supplementary Files [file 41467_2024_53091_MOESM3_ESM.pdf]

Description of Additional Supplementary Files

File Name: Supplementary Data 1
Description: Demographic characteristics of individuals included in the GWAS of quantitative trait biomarkers. Although the exact number of individuals included varied between traits, demographics are shown for the individuals with available genetic and phenotypic data (40,535) for at least one trait.

File Name: Supplementary Data 2
Description: Overview of quantitative trait phenotype quality control. Columns A - G show the trait name, the units, the final sample size for the phenotype (note that eventual sample size may be slightly smaller where some people failed genotype QC / had missing genotype data), the observed post-QC phenotype mean, SD, minimum, and maximum. Columns H & I show the pre-specific minimum and maximum values used to filter out implausible values. Note that values were included if they were greater than or equal to the minimum, and less than or greater to the maximum. The date of phenotype data QC is shown in column J. Columns K - X show the number of individual readings (i.e. individual test results) at each stage of phenotypic QC. In order, these columns indicate the number of readings initially, after harmonising units, after filtering to those people with non-missing covariate data, after deduplication, after filtering to data from those age > 18 and from before the date of analysis, after filtering on the pre-specific min/max range, after deduplication by time window (i.e. removing duplicate values within a 10-day window), after removal of values on or after the initiation of medication, after restricting to primary care data, and after filtering out outliers >10 standard deviations from the mean on the log10 scale. Subsequent columns show the same QC steps but instead show the number of unique individual participants at each QC step.

File Name: Supplementary Data 3
Description: Overview of GWAS results from the Genes & Health study. Columns show (in order) the trait, the median number of individuals in the GWAS (across all SNPs), the total number of SNPs, the number of independent GWAS-significant loci, the units, the lambda (genomic inflation factor), and the LD score regression intercept with its standard error. Traits are ordered by N.

File Name: Supplementary Data 4
Description: GWAS summary statistics for significant hits ($P < 1.2e-9$) in the Genes & Health cohort. SNPs are annotated with the nearest gene. Genomic co-ordinates are in hg38. Allele frequencies from gnomAD from the major ancestral groups are shown. Note that only variants which could be matched to a unique rsID and found in gnomAD are shown. GnomAD population codes: AFR = African, AMI = Amish, AMR = Latino/Admixed American, ASJ = Ashkenazi Jewish, EAS = East Asian, FIN = Finnish European, MID = Middle Eastern, NFE = Non-Finnish European, OTH = Other, SAS = South Asian. P values are two-sided P values from the mixed linear model GWAS implemented in REGENIE.

File Name: Supplementary Data 5
Description: Cross-ancestral genetic correlations for quantitative traits, compared between South Asian individuals enrolled in Genes & Health and European individuals enrolled in UK Biobank,

estimated from genome-wide association summary statistics using Popcorn. P values give the two-sided probability of the genetic correlation differing from 1 (i.e. a Z test). Popcorn does not produce estimates for standard error, z score or p value when the estimated correlation is 1.

File Name: Supplementary Data 6
Description: UK Biobank phenotypes used for cross-ancestry comparisons. GWAS summary statistics were obtained from the pan-UKB project. For each trait, the exact name and filename are given. The N for each trait per ancestry varied between traits, and is shown for all 29 traits included in the meta-analysis and fine mapping.

File Name: Supplementary Data 7
Description: Summary statistics for multi-ancestry meta-analysis of all traits. Only SNPs a) achieving study-wide significance for association and b) showing significant ancestral heterogeneity are shown. Study-wide significance for this analysis was set at 1.72x10^-9 (5e-8/29). P values reflect two-sided P values from the meta-analysis.

File Name: Supplementary Data 8
Description: Results from multi-ancestry fine mapping using SuSiEX. All loci showing evidence of ancestral heterogeneity were fine-mapped. Results are shown for each locus and each trait. For each locus, the individual credible sets are listed. For each credible set, the SNP with the maximum causal probability is shown, along with its frequencies, effect estimates, and standard errors across cohorts. For each credible set the causal probability within each ancestral group is shown. The 'locus name' coumn indicates the fine mapping window in hg38 co-ordinates (i.e. 500KB either side of the lead SNP from meta-analysis). Note that some loci began at the start of the chromosome, and these are indicated with a negative value for the start of the window. Credible sets were termed SAS-specific if they had a strong probability of being causal in both Genes & Health and UKB SAS cohorts (>0.8) but not any of the other UKB cohorts (EUR, EAS, AFR). For the 'REF', 'ALT', 'BETA', and 'SE' columns, the REF, ALT, beta, and standard error are shown in order for each of the five populations (GH SAS, UKB EUR, UKB AFR, UKB EAS, and UKB SAS). If a variant was not included in the fine-mapping for that ancestry (e.g. due to MAF < 0.01) these columns show an 'NA'. The 'shared' column indicates whether the causal signal was evident in all populations (i.e. causal probability > 0.8).

File Name: Supplementary Data 9
Description: Results from single-causal variant fine mapping in Genes & Health only at the PIEZO1 locus for HbA1c. For each SNP, the post-hoc probability of being causal is shown. P values are the two-sided P values from the GWAS.
